# Efficacy and Safety of Early Initiation of Sodium–Glucose Co-transporter-2 Inhibitors Following Acute Myocardial Infarction: A Systematic Review and Meta-analysis

**DOI:** 10.17925/EE.2025.21.1.1

**Published:** 2025-02-07

**Authors:** Deep Dutta, Lakshmi Nagendra, ABM Kamrul-Hasan, Kunal Mahajan

**Affiliations:** 1. Department of Endocrinology, CEDAR Super-speciality Healthcare, Dwarka, New Delhi, India; 2. Department of Endocrinology, JSS Medical College, JSS Academy of Higher Education and Research, Mysuru, India; 3. Department of Endocrinology, Mymensingh Medical College, Mymensingh, Bangladesh; 4. Department of Cardiology, Himachal Heart Institute, Mandi, Himachal Pradesh, India

**Keywords:** Dapagliflozin, empagliflozin, meta-analysis, myocardial infarction, safety, sodium–glucose co-transporter-2 inhibitor

## Abstract

**Background.:**

Sodium–glucose co-transporter-2 inhibitors (SGLT2i) are the preferred agents for managing type 2 diabetes in patients with established atherosclerotic cardiovascular disease and for reducing hospitalization for heart failure (HHF) in patients with heart failure with reduced and preserved ejection fraction. We undertook this meta-analysis, as, to date, no meta-analysis has holistically analysed the potential benefits and safety of SGLT2i in patients with acute myocardial infarction (MI).

**Methods.:**

Electronic databases were searched for randomized controlled trials (RCTs) involving patients with MI who received SGLT2i in the intervention arm (initiated within 2 weeks of the index event) and placebo/active comparator in the control arm. The primary outcome was to evaluate the impact on cardiovascular death, all-cause death and HHF. The secondary outcomes were to evaluate the impact on echocardiographic parameters, N-terminal pro-b-type natriuretic peptide (NT-proBNP), high-sensitivity C-reactive protein, MI, stroke, all-cause hospitalization and safety issues.

**Results.:**

From initially screened 8,922 articles, data from 6 RCTs were analysed (7,409 patients). Early initiation of SGLT2i following MI was associated with significantly lower future HHF (odds ratio [OR]: 0.75; 95% confidence interval [CI]: 0.62–0.90; p=0.002; *I*^2^=0%) and significantly higher left-ventricular ejection fraction (mean difference [MD]: 1.65%; 95% CI: 0.34–2.96; p=0.01; *I*^2^=0%) compared with placebo. Compared with placebo, SGLT2i following MI had no beneficial impact on cardiovascular deaths (OR: 1.04; 95% CI: 0.83–1.30; p=0.76; *I*^2^=0%), all-cause mortality (OR: 1.00; 95% CI: 0.82–1.21; p=0.98; *I*^2^=0%), stroke (OR: 0.58; 95% CI: 0.26–1.27; p=0.17), all-cause hospitalization (OR: 1.13; 95% CI: 0.97–1.32; p=0.11; *I*^2^=0%) and percentage change in NT-proBNP (MD: 1.18%; 95% CI: -9.78 to 12.14; p=0.83; *I*^2^=52%). SGLT2i were well tolerated without increased ketoacidosis, acute renal failure or hepatic injury.

**Conclusion.:**

Early initiation of SGLT2i in acute MI is safe, well tolerated and associated with a reduction in HHF.

## Article Highlights

Early use of sodium–glucose co-transporter-2 inhibitors following myocardial infarction was associated with the following factors:

- Lower hospitalization for heart failure (odds ratio [OR]: 0.75; 95% confidence interval [CI]: 0.62–0.90; p=0.002).
- Similar cardiovascular deaths (OR: 1.04; 95% CI: 0.83–1.30; p=0.76).
- Similar all-cause mortality (OR: 1.00; 95% CI: 0.82–1.21; p=0.98).
- Similar risks of ketoacidosis, acute renal failure or hepatic injury.

Early and timely percutaneous coronary intervention (PCI) therapies, effective anti-platelet therapy along with aggressive early lipid lowering with high-i ntensity statins and other lipid mediations are the current cornerstones for improving short- and long-term outcomes in patients with acute myocardial infarction (AMI).^1^ However, despite these advances, a significant amount of residual cardiovascular (CV) risk remains in these patients for a recurrent CV event, especially in the initial few weeks of the index event. Sodium–glucose co- transporter-2 inhibitors (SGLT2i) are considered to be the preferred agents for managing type 2 diabetes (T2D) in patients with established atherosclerotic cardiovascular disease (ASCVD) and those with multiple risk factors for ASCVD.^2,3^ In addition, SGLT2i have established themselves as the preferred agents for reducing hospitalization for heart failure (HHF) in patients with heart failure with reduced and preserved ejection fraction, regardless of their underlying glycaemic status.^2–4^ SGLT2i have demonstrated themselves to improve a broad range of CV outcomes, especially CV death and HHF in different randomized controlled trials (RCTs) and meta-analyses.^3–5^

Recently, several RCTs have been published evaluating the role of SGLT2i in myocardial infarction (MI).^6–9^ Traditionally, the use of SGLT2i, in general, has been avoided during acute illness (infections, surgery or acute events such as AMI) due to safety concerns primarily related to the increased risk of euglycaemic ketosis.^10^ In addition, the effectiveness of a medicine in improving CV and mortality outcomes in patients with stable ASCVD and chronic heart failure does not guarantee its efficacy in AMI. A prime example is sacubitril-valsartan, which reduces CV deaths and HHF in patients with chronic heart failure with reduced ejection fraction but not when used in the setting of AMI (Prospective ARNI versus ACE Inhibitor Trial to Determine Superiority in Reducing Heart Failure Events after MI [PARADISE-MI trial]; ClinicalTrials. gov identifier: NCT02924727).^11,12^ This makes it even more important to study SGLT2i in AMI, despite their proven efficacy in chronic heart failure. A literature review revealed that no meta-analysis is available that has holistically analysed and summarized the clinical efficacy and safety of SGLT2i following MI. Hence, the aim of this systematic review and meta-analysis (SRM) was to evaluate the efficacy and safety of SGLT2i in MI.

### Methods

#### Methodology

This meta-analysis followed the Preferred Reporting Items for Systematic Reviews and Meta-Analyses checklists and the procedures described in the Cochrane Handbook for Systematic Reviews of Interventions.^13,14^ The SRM was registered with PROSPERO (CRD42024533973), and the protocol summary is accessible online. All RCTs published till March 2024 were considered for this meta-analysis. As ethical approval already exists for the individual studies included in the meta-analysis, no separate approval was required for this study.

Population, Intervention, Comparison, Outcomes and Study design was used as a framework to formulate eligibility criteria for the clinical trials in this SRM. The patient population (P) consisted of patients with MI; the intervention (I) was the use of SGLT2i along with the standard therapy for managing MI; the comparison or control (C) involved patients either on placebo or any other medication over the background standard therapy for MI; the outcomes (O) evaluated included all-cause death/mortality, CV death, HHF, stroke, recurrence of MI, changes in N-terminal pro-b-type natriuretic peptide (NT-proBNP), weight, echocardiography parameters and any adverse effects noted; and RCTs were considered as the study type (S) for inclusion. This study comprised RCTs with study individuals aged at least 18 years. Only those RCTs were considered for this meta-analysis where SGLT2i was initiated within 2 weeks of the index MI event.

The primary outcome was to evaluate the changes in CV death, all-cause death/mortality and HHF. The secondary outcomes of this study were to evaluate the alterations in echocardiographic parameters (left ventricular ejection fraction [LVEF]), NT-proBNP, high-sensitivity C-reactive protein (hs-CRP), occurrence of stroke, recurrence of MI, all-cause hospitalization and safety issues such as changes in weight, occurrence of ketoacidosis, acute renal failure and hepatic injury. Sub-group analysis was performed based on whether the control group received an active comparator (active control group) or a placebo (passive control group).

#### Search method for identifying studies

Several databases and registers, including MEDLINE (via PubMed), Scopus, Cochrane Central Register and ClinicalTrials. gov, were systematically searched. The search covered these sources from their commencement to 30 March 2024. The search terms were applied to titles only; the search technique followed a Boolean approach using the terms ‘SGLT2’ OR ‘sodium glucose co-transporter-2 inhibitor’ OR ‘dapagliflozin’ OR ‘empagliflozin’ OR ‘canagliflozin’ OR ‘ertugliflozin’ OR ‘sotagliflozin’ AND ‘myocardial infarction’.

Every recently published or unpublished clinical study in English was searched exhaustively and carefully. This search involved looking through pertinent publications and references found in the clinical trials included in the present work.

#### Data extraction, study selection, measurement of treatment effects and data synthesis

Four review authors independently conducted data extraction using standardized data extraction forms, with details provided elsewhere.^15,16^ The handling of missing data has also been elaborated upon in the same source.^15,16^ RevMan Web 2024 version was used for comparing the mean difference (MD) of the different primary and secondary outcomes between the SGLT2i and the control groups of the included studies. Random effects analysis models were chosen to address the anticipated heterogeneity due to variations in population characteristics and trial lengths. The inverse variance statistical method was applied for all instances. The meta-analysis encompassed forest plots that integrated data from a minimum of two trials. A significance level of p<0.05 was used.

#### Assessment of risk of bias in the included studies

Three authors independently assessed the risk of bias (ROB) using the ROB assessment tool in Review Manager (RevMan) Web Version 2024 (The Cochrane Collaboration, Oxford, UK, 2024) software. ROB assessment was performed under the following headings: adequate sequence generation (selection bias); adequate allocation concealment (selection bias); adequate prevention of knowledge of allocated interventions during the study; blinding of participants and personnel (performance bias); blinding of outcome assessors (detection bias); incomplete outcome data (attrition bias); and freedom from selective outcome reporting (reporting bias). Involvement of pharmaceutical organizations in the funding, conducting the study and preparing the draft was considered to be high ROB under other bias sub-headings.

#### Assessment of heterogeneity

The assessment of heterogeneity was initially conducted by studying forest plots. Subsequently, a *χ*^2^ test was performed using N-1 degrees of freedom and a significance level of 0.05 to determine the statistical significance. The *I*^2^ test was also used in the subsequent analysis.^14^ The specifics of understanding *I*^2^ values have already been explained in depth elsewhere.^15,16^

#### Grading of the results

The Grading of Recommendations Assessment, Development and Evaluation methodology was used to determine the quality of evidence about each meta-analysis outcome.^17,18^ The details of generating the summary of findings (SoF) table and judging the quality of evidence as ‘high’, ‘moderate’, ‘low’ or ‘very low’ have been previously reported.^15,16^

**Figure 1:**
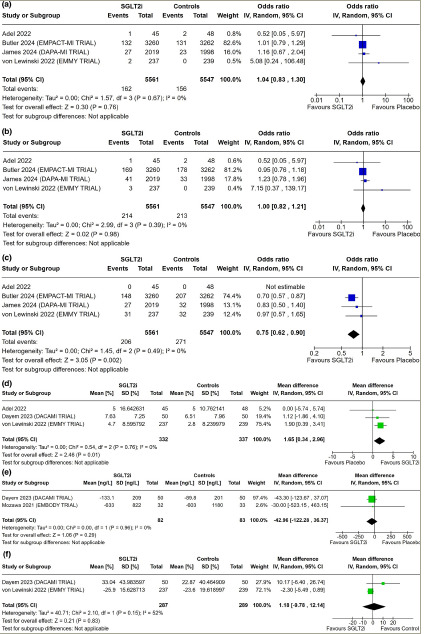
Forest plot highlighting the impact of early initiation of sodium–glucose co-transporter-2 inhibitors in patients with myocardial infarction

*(a) Cardiovascular death; (b) all-cause death/mortality; (c) hospitalization for heart failure; (d) LVEF; (e) NT-proBNP and (f) percentage change in NT-proBNP from baseline.*

*CI = confidence interval; df = degrees of freedom; IV = inverse variance; LVEF = left ventricular ejection fraction; NT-proBNP = N-terminal pro-b-type natriuretic peptide; SD = standard deviation; SGLT2i = sodium–glucose co-transporter-2 inhibitors.*

### Results

This SRM was done as per the preregistered protocol with PROSPERO without any deviation (CRD42024533973). A total of 8,922 articles were found after the initial search (*Figure 1*). Four hundred and eighty duplicates were removed following the screening of the titles, and the search was reduced to 106 articles. After further review of these 106 abstracts, the search was reduced to 12 studies, which were then evaluated in detail for inclusion in this meta-analysis (*Supplementary Material 1*). Eight articles presenting data from six different RCTs (7,409 patients) that fulfilled all criteria were analysed in this meta-analysis.^6–9,19–22^

The study by James et al. was a double-blinded RCT comparing 1-year outcomes of dapagliflozin 10 mg/day with placebo initiated in patients with AMI within 10 days of the index event (Dapaglifozin Effects on Cradiometabolic Outcomes in Patients with an Acute Heart Attack [DAPA-MI]; ClinicalTrials. gov identifier: NCT04564742).^6^ The study by Butler et al. was a double-blinded RCT comparing outcomes in patients receiving empagliflozin 10 mg/day with placebo when initiated with 14 days of AMI, having a mean follow-up of around 18 months (Empagliflozin after Acute Myocardial Infarction [EMPACT-MI]: A Study to Test Whether Empagliflozin Can Lower the Risk of Heart Failure and Death in People Who Had a Heart Attack [Myocardial Infarction]; ClinicalTrials. gov identifier: NCT04509674).^7^ The study by Dayem et al. was a double-blinded RCT comparing 12-week outcomes of the impact on NT-proBNP and echocardiography parameters after the initiation of dapagliflozin 10 mg/day with placebo in patients with AMI (Impact of Dapagliflozin on Cardiac Function Following Anterior Myocardial Infarction in Non-diabetic Patients [DACAMI trial]; ClinicalTrials. gov identifier: NCT05424315).^8^ In the DACAMI trial, dapagliflozin was started within 72 h of ST elevation myocardial infarction.^8^ The study by von Lewinski et al. was a double-blinded RCT evaluating 26-week outcomes of the impact on NT-proBNP and echocardiographic parameters after the initiation of empagliflozin 10 mg/day with placebo, initiated within 72 h of PCI in patients with AMI (Empagliflozin in Acute Myocardial Infarction [EMMY trial]; ClinicalTrials. gov identifier: NCT03087773).^9^ Benedikt et al. studied changes in inflammatory markers with empagliflozin therapy in the same cohort of patients with MI from the EMMY trial.^19^ Therefore, the results from this article have been analysed under von Lewinski et al. in this SRM. Sourij et al. analysed the gender differences in response to empagliflozin therapy after AMI in the cohort of patients of the EMMY trial.^20^ The study by Mozawa et al. was a double-blinded RCT evaluating the impact of empagliflozin 10 mg/day compared with placebo initiated within 2 weeks of AMI in Japanese patients (Effects of Empagliflozin versus Placebo on Cardiac Sympathetic Activity in Acute Myocardial Infarction Patients with Type 2 Diabetes Mellitus [EMBODY trial]: UMIN000030158]).^21^ The article by Hoshika et al. was from the same cohort of patients in the EMBODY trial.^22^ Therefore, the results from this study have been presented under Mozawa et al. in this SRM. The study by Adel et al. evaluated the impact of empagliflozin 10 mg/day compared with placebo in improving CV outcomes in patients with diabetes with acute coronary syndrome (ACS) after PCI.^23^ The details of the studies included in this SRM have been elaborated in *Table 1*.^6–9,19,20^

SOdium–glucose CO-transporter inhibition in patients with newly detected Glucose Abnormalities and a recent Myocardial Infarction (SOCOGAMI, EudraCT number: 2015-004571-73) was a randomized, double-blind, placebo-controlled trial, which was excluded from this SRM, as it involved patients with AMI or unstable angina pectoris in the last 6 months.^24,25^ The study by Khiali et al. was excluded from this analysis, although they initiated empagliflozin alone or in combination with colchicine, within 72 h of MI, as they did not evaluate the primary and secondary outcomes evaluated in this SRM.^26^ They looked at changes in echocardiographic parameters and systemic inflammatory markers after 12 weeks of empagliflozin and/or colchicine use following AMI.^26^ The RCT by Karetnikova et al. evaluated the role of empagliflozin in patients undergoing PCI for coronary artery disease (CAD).^27^ However, this RCT was excluded from our analysis because the elective PCI was done in stable patients with CAD rather than in the setting of AMI.^27^ In addition, empagliflozin was initiated 1 month before the elective PCI.^27^ Therefore, this RCT did not fulfil our inclusion and exclusion criteria.

#### Risk of bias in the included studies

The summaries of ROB of the six RCTs included in this SRM have been elaborated in *Supplementary Material 2a and b.* Random sequence generation, allocation concealment (selection bias), blinding of participants and personnel (performance bias), blinding of outcome assessment (detection bias), attrition bias and reporting bias were judged to be at low ROB in all six studies (100%). Source of funding, especially pharmaceutical, authors from the pharmaceutical organizations and conflict of interests were looked into the ‘other bias’ section. Other bias was judged to be at low risk in two out of six RCTs (33.33%) (*Supplementary Material 2a and b*).

#### Effect of sodium–glucose co-trasnporter-2 inhibitors on primary outcomes

##### Cardiovascular death, all-cause death/mortality and hospitalization for heart failure

Data from four studies involving 11,108 patients with AMI were analysed to find out the impact of early initiation of SGLT2i following MI on CV death, all-cause death and HHF. CV deaths following AMI were similar in patients initiated on SGLT2i compared with placebo (odds ratio [OR]: 1.04; 95% confidence interval [CI]: 0.83–1.30; p=0.76; *I*^2^=0% [low heterogeneity]; *Figure 1a*). All-cause mortality was also similar in patients receiving SGLT2i compared with placebo, following AMI (OR: 1.00; 95% CI: 0.82–1.21; p=0.98; *I*^2^=0% [low heterogeneity]; *Figure 1b*). HHF was significantly lower in patients who had early initiation of SGLT2i following MI compared with placebo (OR: 0.75; 95% CI: 0.62–0.90; p=0.002; *I*^2^=0% [low heterogeneity]; *Figure 1c*).

#### Effect of sodium–glucose co-trasnporter-2 inhibitors on secondary outcomes

##### Left ventricular ejection fraction

Data from three studies involving 669 patients with AMI were analysed to find out the impact of early initiation of SGLT2i following MI on LVEF in echocardiography. Patients initiated on SGLT2i had significantly higher LVEF compared with those on placebo (MD: 1.65%; 95% CI: 0.34–2.96; p=0.01; *I*^2^=0% [low heterogeneity]; *Figure 1d*).

#### N-terminal pro-b-type natriuretic peptide

Data from two studies (165 patients) on AMI were analysed to find out the impact of SGLT2i on circulating NT-proBNP levels. Changes in the absolute value of NT-proBNP (MD: -42.96 ng/L; 95% CI: -122.28 to 36.37; p=0.29; *I*^2^=0% [low heterogeneity]; *Figure 1e*) were similar in patients receiving SGLT2i compared with those receiving placebo. Data from two studies (576 patients) on AMI were analysed to find out the impact of SGLT2i on the percentage change in circulating levels of NT-proBNP compared with baseline. Percentage change in NT-proBNP was similar in patients receiving SGLT2i compared with placebo (MD: 1.18%; 95% CI: -9.78 to 12.14; p=0.83; *I*^2^=52% [moderate heterogeneity]; *Figure 1f*).

#### High-sensitivity C-reactive protein

**Figure 2:**
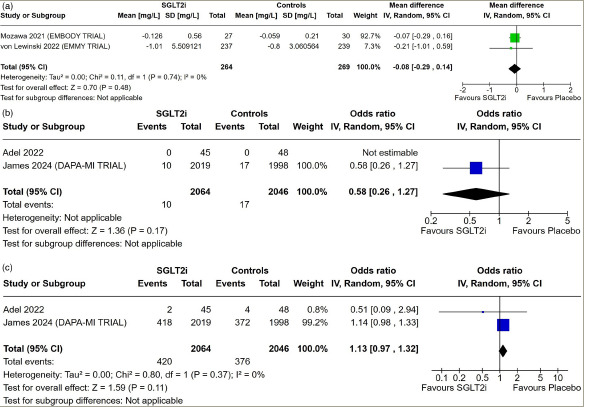
Forest plot highlighting the impact of early initiation of sodium–glucose co-transporter-2 inhibitors in patients with myocardial infarction

Data from two studies having 533 patients with AMI were analysed to find out the impact of SGLT2i on circulating inflammatory marker hs-CRP. hs-CRP levels were similar in patients receiving SGLT2i compared with those receiving placebo (MD: -0.08 mg/L; 95% CI: -0.29 to 0.14; p=0.48; *I*^2^=0% [low heterogeneity]; *Figure 2a*).

#### Stroke, all-cause hospitalization and myocardial infarction

Data from two studies involving 4,110 patients with AMI were analysed to find out the impact of early initiation of SGLT2i following MI on the occurrence of stroke and all-cause hospitalization. Stroke (OR: 0.58; 95% CI: 0.26–1.27; p=0.17; *Figure 2b*) and all-cause hospitalization (OR: 1.13; 95% CI: 0.97–1.32; p=0.11; *I*^2^=0% [low heterogeneity]; *Figure 2c*) following AMI were similar in patients on SGLT2i compared with placebo. Data from one study were available analysing the occurrence of a recurrent event of MI following the use of SGLT2i after an index AMI. Recurrence of MI following AMI was similar in patients on SGLT2i compared with placebo (OR: 1.12; 95% CI: 0.72–1.73; p=0.61; *I*^2^=0%; DAPA-MI trial).^6^

**Table 1:** Baseline characteristics of patients in the randomized controlled trials analysed in this systematic review and meta-analysis^6–9^,^19^,^20^

		Adel 2022^20^		Butler 2024 (EMPACT-MI trial)^7^		Dayem 2023 (DACAMI trial)^8^		James 2024 (DAPA-MI trial)^6^		Mozawa 2021 (EMBODY trial)^19^		von Lewinski 2022 (EMMY trial)^9^	
Parameter		Empagliflozin (n=45)	Control/placebo (n=48)	Empagliflozin (n=3,260)	Control/placebo (n=3,262)	Dapagliflozin (n=50)	Control (n=50)	Dapagliflozin (n=2,019)	Control/placebo (n=1,998)	Empagliflozin (n=46	Control/placebo (n=50)	Empagliflozin (n=237)	Control/placebo (n =239)
Age (years)		55 (45.5-64)	57 (50-66.75)	63.6 ± 11.0	63.7 ± 10.8	55.24 ± 13.2	56.70 ± 11.5	63.0 ± 11.06	62.8 ± 10.64	63.9 (10.4)	64.6 ± 11.6	57 (52-64)	57 (52-65)
Male sex (%)		60.0	60.4	75.1	75.1	84	82	80.8	79.0	82.6	78	82	82
Weight (kg)		75 (67.5-84.5)	69.5 (65-83.75)	-	-	-	-	85.5 ± 15.87	85.5 ± 16.54	70.1 ± 13.7	68.1 ± 14.4	-	-
BMI (kg/m^2^)		-	-	28.1 ± 5	28.1 ± 5	29.96 ± 4.9	30.13 ±4.6	-	-	25.2 ±3.7	25.2 ± 4.1	27.7 (25.3-30.3)	27.2 (24.9-30.2)
SBP (mm Hg)		130 (116.25-150)	130 (116.25-140)	120.3 (14.6)	120.5 (15.2)	-	-	119.1 ± 16.23	118.7 ± 16.62	-	-	125 (116-131)	125 (118-131)
eGFR (mL/min/1.73 m^2^	72 (61-83)	76 (61.25-81)	77.5 (62.2-91.0)	78.0 (61.7-91.4)	82.61 ± 14.31	85.49 ± 13.49	83.5 ± 17.12	83.4 ± 16.91	64.60 ± 14.95	66.14 ± 15.72	92 (78-101)	91 (78-102)
HbA1c(%)		7.8 (7.2-8.45)	7.8 (7.1-8.05)	-	-	-	-	5.7 ± 0.58	5.7 ± 0.51	6.82 ± 1.00	6.89 ± 0.92	5.60 (5.40-6.00)	5.70 (5.40-6.00)
Index Ml (%)	STEMI	60	50	75	73.6	-	-	72.6	71.5	-	-	-	-
	NSTEMI	4.4	8.3	25	26.4	-	-	27.4	28.5	-	-	-	-
Previous cardiovascular history (%)	Ml			11.9	14.1	12	14	8.8	9.5	-	-	5.9	3.8
	Stroke	2.2	4.2	-	-	0	0	2.3	2.5	15.2	22	2.1	0.4
	AF	-	-	11.0	11.1	-	-	-	-	-	-		
	T2D	100	100	31.7	32.1	-	-	0	0	100	100	13	14
	HT	57.8	66.7	69.4	69.8	64	58	-	-	82.6	78.0	39	45
	PAD	-	-	5.3	5.5	-	-	-	-	-	-	-	-
Unstable angina (%)		35.6	43.8	0	0	-	-	0	0	-	-	-	-
Patient characteristics		Inclusion criteria: adults over 18 years with a prior diagnosis of T2D and ACS, including ST elevation Ml, non-ST elevation Ml or unstable angina. Exclusion criteria: DKA, urinary and genital infections,T1D, severe liver failure, malignancy, eGFR <30 mL/min/1.73 m^2^ and nonadherence		Inclusion criteria: adults over 18 years hospitalized with acute Ml within 14 days before randomization, presenting evidence of either a newly developed LVEF <45% or signs/ symptoms of congestion necessitating treatment. Exclusion criteria: previous heart failure diagnosis and use of SGLT2i		Inclusion criteria: patients with anterior STEMI, LVEF <50% and successful pPGI. Exclusion criteria: patients with diabetes, prior HF, cardiotoxic medication, haemoglobinopathies, chronic organ damage, existing SGLT2i use, need for additional anticoagulation or contraindications for dapagliflozin		Inclusion criteria: stable adults over 18 years hospitalized for acute Ml and with LV systolic dysfunction or Q-wave Ml on ECG. Exclusion criteria: established diabetes, symptomatic HF with reduced LVEF <40% and current SGLT2i treatment		Inclusion criteria: adults over 20 years with diagnosed T2D according to Japanese guidelines and patients within 2-12 weeks after the onset of acute ML Exclusion criteria: T1D, persistent AF, use of insulin, GLP1 RA or high-dose sulfonylurea, HbA1G >10%, recent DKA or coma, renal dysfunction (eGFR <45 mL/min/1.73 m^2^), NYHA functional class IV heart failure, pregnancy or breastfeeding and contraindications to empagliflozin		Inclusion criteria: patients aged 18-80 years with confirmed acute large Ml (GK >800 IU/L), high troponin T (or I) >10x upper limit, and eGFR >45 mL/min/1.73 m^2^. Exclusion criteria: patients with non T2D, pH <7.32, haemodynamic instability, acute UTI or genital infection, current or recent SGLT2i treatment	
Duration of study (weeks)		24		24		12		48		24		26	
Key outcomes	-		There was no difference in the incidence of hypoglycaemia or lower limb amputations		None of the patients in the study group receiving dapagliflozin encountered genitourinary infections or hypoglycaemic episodes		There was no increase in serious adverse events related to adverse reactions that could potentially be associated with SGLT2I, such as hypovolaemia, hypotension, amputations or genital infections		-		Incidence of genital infections and showed no significant difference between the empagliflozin and placebo. No amputations or severe hypoglycaemic episodes were reported. While beta-hydroxybutyrate concentrations increased significantly more in the empagliflozin group compared with placebo, there was no difference in the occurrence of DKA	

*All continuous variables have been expressed either as mean ± standard deviation or median (interquartile range).*

*ACS = acute coronary syndrome; AF = atrial fibrillation; BMI = body mass index; CK = creatine kinase; DKA = diabetic ketoacidosis; ECG = electrocardiogram; eGFR = estimated glomerular filtration rate;GLP1RA = glucagon-like peptide-1 receptor agonist; HbAIC = haemoglobin A1C; HF = heart failure; HT = hypertension; LV = left ventricular; LVEF = left ventricular ejection fraction; Ml = myocardial infarction; NSTEMI = non-ST elevation myocardial infarction; NYHA = New York Heart Association; FAD = peripheral artery disease; pPCI = primary percutaneous coronary intervention; SBP = systolic blood pressure; SGEf2i = sodium-glucose co-trasnporter-2 inhibitors; SfEMI = ST elevation myocardial infarction; T1D = type 1 diabetes; T2D = type 2 diabetes; UTI = urinary tract infection.*

*(a) hs- CRP; (b) stroke and (c) all- cause hospitalization.*

*CI = confidence interval; df = degrees of freedom; hs- CRP = high-sensitivity C- reactive protein; IV = inverse variance; SD = standard deviation; SGLT2i = sodium–glucose co-trasnporter-2 inhibitors.*

#### Safety

##### Weight

**Figure 3:**
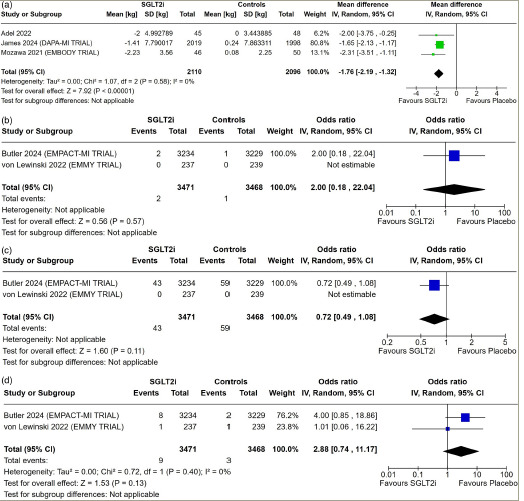
Forest plot highlighting the impact of early initiation of sodium–glucose co-transporter-2 inhibitors in patients with myocardial infarction

Data from three studies involving 4,206 patients with AMI were analysed to find out the impact of early initiation of SGLT2i following MI on body weight. Patients receiving SGLT2i had significantly lower body weight compared with placebo (MD: -1.76 kg; 95% CI: -2.19 to -1.32]; p<0.001; *I*^2^=0% [low heterogeneity]; *Figure 3a*).

#### Ketoacidosis, acute renal failure and hepatic injury

Data from two studies involving 6,939 patients with AMI were analysed to find out the impact of early initiation of SGLT2i following MI on the occurrence of ketoacidosis, acute renal failure and hepatic injury. The occurrence of ketoacidosis (OR: 2.00; 95% CI: 0.18–22.04; p=0.57; *Figure 3b*), acute renal failure (OR: 0.72; 95% CI: 0.49–1.08; p=0.11; *Figure 3c*) and hepatic injury (OR: 2.88; 95% CI: 0.74–11.17; p=0.13; *I*^2^=0% [low heterogeneity]; *Figure 3d*) was similar in patients receiving SGLT2i compared with placebo.

Funnel plots were plotted to evaluate the presence of publication bias and have been elaborated in *Supplementary Material 3.* All the key outcomes had low publication bias. The SoF of some of the major outcomes of this SRM has been elaborated in *Table 2*. All the key outcomes of this SRM had a high grade of evidence.

## Discussion

In the different cardiovascular outcome trials (CVOTs) in patients with T2D, only empagliflozin and canagliflozin have demonstrated superiority in reducing 3- point major adverse CV events (3P MACE; CV mortality, nonfatal MI and nonfatal stroke) compared with placebo.^28,29^ The same has not been seen in CVOTs with dapagliflozin, ertugliflozin and sotagliflozin, highlighting the heterogeneity in outcomes across different SGLT2i.^30–32^ The heterogeneity seen with SGLT2is in terms of CVOT outcomes may be related to trial populations and study designs rather than the individual molecules. In a meta-analysis of CVOTs of different SGLT2i in T2D, a significant reduction in CV death and all-cause mortality has been documented.^33^ Data with regard to the reduction of HHF and heart failure-related deaths with the use of SGLT2i in patients with or without diabetes are more homogeneous and robust across the different SGLT2i.^4^

SGLT2 inhibitors may have some protective benefit in reducing contrast-i nduced acute kidney injury events in patients with ACS undergoing PCI.^34^ Patients with AMI tend to be in a more critical condition and have a different metabolic milieu compared with stable patients living with T2D seen in the outpatient departments. Whether SGLT2i can replicate the same CV benefits in patients with MI as has been seen in patients living with T2D is not known.^34^

*(a) Body weight; (b) ketoacidosis; (c) acute kidney injury and (d) hepatic injury.*

*CI = confidence interval; df = degrees of freedom; IV = inverse variance; SD = standard deviation; SGLT2i = sodium–glucose co-trasnporter-2 inhibitors.*

This is the first SRM to highlight the efficacy and safety of early initiation of SGLT2i following AMI. The initiation of SGLT2i within 2 weeks of AMI was associated with significantly reduced future risk of HHF, without any additional beneficial impact on CV mortality, all-cause mortality, stroke and all-cause hospitalization. No significant improvement in circulating levels of NT-pro-BNP was noted. In addition, no improvement in systematic inflammation (hs-CRP) was noted. The use of SGLT2i following MI was well tolerated, without any increased occurrence of ketoacidosis, acute renal failure and hepatic injury. A mild but statistically significant reduction in body weight was noted.

This SRM highlights that the benefits of using SGLT2i in patients with MI are restricted to improving heart failure outcomes, without any impact on ASCVD and mortality. Therefore, the results are much more tempered compared with those seen in patients with T2D with ASCVD. Mukhopadhyay et al., in their meta-analysis of CVOTs of SGLT2i in T2D, highlighted that SGLT2i reduces MACE without significantly reducing the incidence of MI or stroke (fatal and nonfatal), probably implicating mechanisms unrelated to anti-atherogenic effects.^35^ It is now increasingly being considered that the reduction in CV death and all-cause mortality with the use of SGLT2i in T2D, without any significant reduction in MI and stroke, may be due to non-atherosclerotic mechanisms such as reduction in heart failure-related events, sudden cardiac death and arrhythmias.^35^ The outcomes of this SRM in patients with MI sync with the evolving understanding of the predominantly vascular non-atherosclerotic mechanism of action of SGLT2i in improving cardiac outcomes. In patients with MI, early use of SGLT2i results in predominantly vascular benefits of reduction in hospital admissions for heart failure, without any beneficial impact on stroke, CV mortality and all-cause mortality. Another reason for the tempered results with SGLT2i use in AMI, as seen in this SRM, may be because the heart failure seen in the setting of AMI is often transient. It results from myocardial stunning, neurohumoral activation and systemic inflammation, which are reversed to a great extent following prompt re-vascularization.^36^

Another class of medication, which has played a major role in improving CV outcomes in patients with diabetes and stable established ASCVD, is glucagon-l ike peptide-1 receptor agonists (GLP1RAs).^37^

**Table 2:** Summary of the findings of the key outcomes of this systematic review and meta-a nalysis evaluating the role of sodium–glucose co-transporter-2 inhibitors in acute myocardial infarction

	Anticipated absolute effects^*^ (95% CI)				
Outcomes	Risk with placebo in the MI group	Risk with SGLT2i in the MI group	Relative effect (95% CI)	No. of participants (studies)	Certainty of the evidence (GRADE)
**CV death**	28 per 1,000	**29 per 1,000** (23-36)	**OR 1.04** (0.83-1.30)	11,108 (four RCTs)	⨁⨁⨁⨁ High
**All-cause death**	38 per 1,000	**38 per 1,000** (32-46)	**OR 1.00** (0.82-1.21)	11,108 (four RCTs)	⨁⨁⨁⨁ High
**Hospitalization for heart failure**	49 per 1,000	**37 per 1,000** (31-44)	**OR 0.75** (0.62-0.90)	11,108 (four RCTs)	⨁⨁⨁⨁ High
**All-cause hospitalization**	184 per 1,000	**203 per 1,000** (179-229)	**OR 1.13** (0.97-1.32)	4,110 (two RCTs)	⨁⨁⨁⨁ High
**Acute renal failure**	17 per 1,000	**12 per 1,000** (8-18)	**OR 0.72** (0.49-1.08)	6,939 (two RCTs)	⨁⨁⨁⨁ High
**Hepatic injury**	1 per 1,000	**2 per 1,000** (1-10)	**OR 2.88** (0.74-11.17)	6,939 (two RCTs)	⨁⨁⨁⨁ High
**Ketoacidosis**	0 per 1,000	**1 per 1,000** (0-6)	**OR 2.00** (0.18-22.04)	6,939 (two RCTs)	⨁⨁⨁⨁ High

*The risk in the intervention group (and its 95% CI) is based on the assumed risk in the comparison group and the relative effect of the intervention (and its 95% CI).

*GRADE Working Group grades of evidence – high certainty: we are very confident that the true effect lies close to that of the estimate of the effect. Moderate certainty: we are moderately confident in the effect estimate; the true effect is likely to be close to the estimate of the effect, but there is a possibility that it is substantially different. Low certainty: our confidence in the effect estimate is limited; the true effect may be substantially different from the estimate of the effect. Very low certainty: we have very little confidence in the effect estimate; the true effect is likely to be substantially different from the estimate of the effect.*

*CI = confidence interval; CV = cardiovascular; GRADE = Grading of Recommendations Assessment, Development and Evaluation; MI = myocardial infarction; OR = odds ratio; RCT = randomized controlled trial; SGLT2i = sodium–glucose co-transporter-2 inhibitors.*

In a meta-analysis of data from six RCTs involving patients with AMI undergoing PCI, GLP1RA treatment was associated with improvement in the LVEF along with a reduction in the infarct size, without any significant reduction in CV events.^38^ Therefore, the outcomes of the use of GLP1RAs in the setting of AMI may be considered to be more tempered compared with the use in stable patients with ASCVD. As suggested by Karakasis et al*.*, one reason may be the lack of dedicated CVOTs with GLP1RA in the setting of ACS; therefore, no solid evidence regarding their true CV efficacy on surrogate endpoints can be generated.^37^ Thus, there remains an urgent need for dedicated studies evaluating the combination therapy of SGLT2i and GLP1RAs in patients with ACS, unstable angina and MI with non-obstructive coronaries. This combination therapy offers a dual beneficial impact on inflammation as well as on endothelial dysfunction.^37^

From this SRM, it is interesting to consider that the reduction in HHF with the use of SGLT2i in MI was not associated with a significant reduction in circulating levels of NT-proBNP, a commonly accepted serologic measure of heart failure. This may primarily be due to the small number of patients evaluated with data being available from three different RCTs only. A limitation of the current SRM is that the analysis was done on extracted summary data of the published RCTs, rather than individual patient data. Only dapagliflozin and empagliflozin have been evaluated in AMI. Data from other SGLT2i are not available. The actual number of different types of CV events in the different RCTs was relatively small. The follow-up duration was short in three of the six RCTs analysed (*Table 1*). Additionally, outcome data of all the variables analysed were not available from all the RCTs analysed.

Our SRM provides reassuring data on the safety of SGLT2i use in AMI. SGLT2i should be especially used in the setting of AMI when there is a history of chronic heart failure, T2D or chronic kidney disease. This SRM supports the early initiation of SGLT2i in patients with AMI during their stay in the hospital or discharge from the hospital. Delayed initiation of SGLT2i post-AMI has the risk of patients being lost to follow up and thus missing out on the benefits of this class of medication. In conclusion, it may be said that the early use of SGLT2i in AMI is safe, well tolerated and associated with a reduction in HHF.
